# Targeted Deletion of PTEN in Kisspeptin Cells Results in Brain Region- and Sex-Specific Effects on Kisspeptin Expression and Gonadotropin Release

**DOI:** 10.3390/ijms21062107

**Published:** 2020-03-19

**Authors:** Ariel L. Negrón, Guiqin Yu, Ulrich Boehm, Maricedes Acosta-Martínez

**Affiliations:** 1 Graduate Program in Neuroscience, Stony Brook University, Stony Brook, NY 11794, USA; a.negronlopez@gmail.com; 2 Department of Physiology and Biophysics, Stony Brook University, Stony Brook, NY 11794, USA; gui.yu@stonybrook.edu; 3 Experimental Pharmacology, Center for Molecular Signaling (PZMS), Saarland University School of Medicine, 66421 Homburg, Germany; ulrich.boehm@uks.eu

**Keywords:** PTEN, luteinizing hormone, mTOR, kisspeptin, hypothalamus, anteroventral periventricular nucleus, arcuate nucleus

## Abstract

Kisspeptin-expressing neurons in the anteroventral periventricular nucleus (AVPV) and the arcuate nucleus (ARC) of the hypothalamus relay hormonal and metabolic information to gonadotropin-releasing hormone neurons, which in turn regulate pituitary and gonadal function. Phosphatase and tensin homolog (PTEN) blocks phosphatidylinositol 3-kinase (PI3K), a signaling pathway utilized by peripheral factors to transmit their signals. However, whether PTEN signaling in kisspeptin neurons helps to integrate peripheral hormonal cues to regulate gonadotropin release is unknown. To address this question, we generated mice with a kisspeptin cell-specific deletion of *Pten* (Kiss-PTEN KO), and first assessed kisspeptin protein expression and gonadotropin release in these animals. Kiss-PTEN KO mice displayed a profound sex and region-specific kisspeptin neuron hyperthrophy. We detected both kisspeptin neuron hyperthrophy as well as increased kisspeptin fiber densities in the AVPV and ARC of Kiss-PTEN KO females and in the ARC of Kiss-PTEN KO males. Moreover, Kiss-PTEN KO mice showed a reduced gonadotropin release in response to gonadectomy. We also found a hyperactivation of mTOR, a downstream PI3K target and central regulator of cell metabolism, in the AVPV and ARC of Kiss-PTEN KO females but not males. Fasting, known to inhibit hypothalamic kisspeptin expression and luteinizing hormone levels, failed to induce these changes in Kiss-PTEN KO females. We conclude that PTEN signaling regulates kisspeptin protein synthesis in both sexes and that its role as a metabolic signaling molecule in kisspeptin neurons is sex-specific.

## 1. Introduction

The hypothalamic-pituitary-gonadal (HPG) axis integrates both internal and external signals that influence the reproductive fitness of mammals [1]. Gonadotropin-releasing hormone (GnRH) neurons are often described as “the master regulator” of the HPG axis as pulsatile GnRH release stimulates the anterior pituitary to secrete luteinizing hormone (LH) and follicle-stimulating hormone (FSH), which in turn act on the gonads to stimulate the production of sex steroid hormones (i.e., estrogen and testosterone) [1]. However, adult GnRH neurons lack receptors for important signals that modulate gonadotropin release such as leptin and estrogen. Therefore, afferent neurons that are sensitive to changes in these peripheral signals play important roles in regulating GnRH function and gonadotropin release [2]. In this regard, hypothalamic kisspeptin-expressing cells have emerged as major GnRH afferent neurons, which are sensitive to peripheral signals including steroid hormones and metabolic cues [2,3].

The neuropeptide kisspeptin (encoded by the *Kiss1* gene) is a potent stimulator of gonadotropin release via its receptor Kiss1R, which is expressed in GnRH neurons [4]. Kisspeptin is a pivotal modulator of pubertal activation and gonadal maturation as the functional loss of *Kiss1* or its receptor result in delayed puberty and hypogonadotropic hypogonadism [5,6]. In the murine hypothalamus, two distinct kisspeptin nuclei have been described: the anteroventral periventricular (AVPV) nucleus and the arcuate (ARC) nucleus. Sex steroids, such as estradiol (E_2_), positively stimulate *Kiss1* gene and peptide expression in the AVPV, whereas the opposite effect is observed in ARC kisspeptin neurons [7]. This region-specific modulation of kisspeptin function by E_2_ is physiologically relevant as kisspeptin cell-specific estrogen receptor alpha (ERα) signaling is essential for the timing and completion of puberty in females [8], and for E_2_ positive feedback stimulation of ovulation [9]. Additionally, kisspeptin expression is regulated in the ARC, a central hypothalamic nucleus that regulates energy homeostasis, by integrating metabolic cues such as leptin and insulin. As such, metabolic alterations that affect the production of these peripheral metabolic hormones, such as obesity or undernutrition, can impact GnRH and gonadotropin release by hypothalamic kisspeptin neurons [2,3]. However, the intracellular signaling mechanisms that integrate these signals within kisspeptin neurons remain unclear.

The dual specificity phosphatase and tensin homolog (PTEN) is a tumor suppressor gene in charge of mediating key cellular processes during central nervous system (CNS) development, including cell survival, proliferation, and morphology [10,11]. In addition, PTEN participates in maintaining adult CNS homeostasis through its control of synaptic plasticity and neuronal excitability [12]. PTEN acts both as a lipid and protein phosphatase and as a regulator of several signaling cascades, most notably as the direct negative regulator of phosphatidylinositol 3-kinase (PI3K). PI3K mediates insulin and leptin signaling through activation of downstream molecules like the serine/threonine-specific protein kinase, Akt, and the mammalian target of rapamycin (mTOR) [13]. The latter acts as a key cellular energy sensor and downstream effector for PTEN regulation of protein synthesis, cell size, and proliferation [14]. Importantly, pharmacological studies suggest that mTOR signaling plays a role in the interplay between energy status and gonad activation by the hypothalamus during puberty [15]. However, the identity of the neuronal populations in which PTEN signaling controls gonadotropin release and reproduction remains unknown. Investigating PTEN’s function in neuronal populations that are key for the control of puberty and fertility, such as kisspeptin neurons, is therefore needed to better understand the molecular mechanisms by which peripherally derived signals such as sex steroid hormones and metabolic cues control the HPG axis.

To test the hypothesis that PTEN signaling modulates kisspeptin neurons’ capacity to regulate gonadotropic responses to steroid negative feedback and to negative energy balance, we generated mice with a deletion of the *Pten* gene specifically in kisspeptin-expressing cells and analyzed their reproductive phenotype and hypothalamic kisspeptin protein expression. We found that PTEN deletion in kisspeptin cells resulted in a brain region-specific hypertrophy, accompanied by decreased fertility in females and reduced gonadotropin responses to gonadectomy in both sexes. In addition, PTEN deletion resulted in a female-specific hyperactivation of mTOR signaling in AVPV and ARC kisspeptin neurons. PI3K-mTOR hyperactivity was associated with higher hypothalamic kisspeptin protein expression and higher plasma LH levels in fasted females compared to controls. Our results shed light on the molecular signaling mechanisms that are involved in defining kisspeptin cell morphology and protein synthesis, with PTEN and its downstream signaling target, mTOR, contributing to these cellular processes.

## 2. Results

### 2.1. Validation of Kiss-PTEN KO

**Figure 1:**
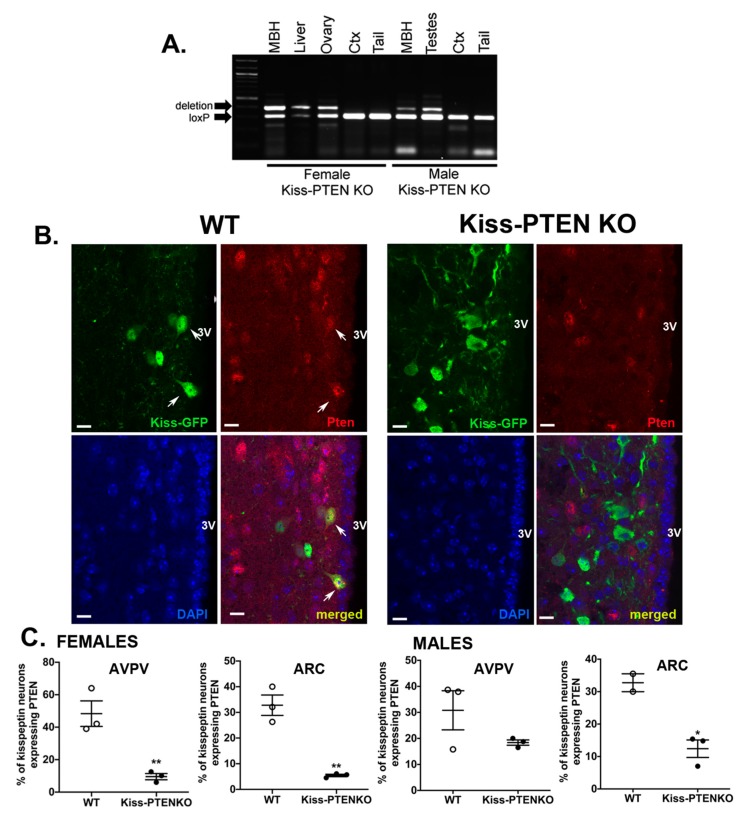
Phosphatase and tensin homolog (PTEN) deletion is restricted to kisspeptin-expressing tissues and neurons in Kiss-PTEN KO mice. (**A**) Representative PCR analyses showing deletion of *Pten* exon 5 (404 bp product) in the mediobasal hypothalamus (MBH), liver, and gonads, whereas no deletion is observed in the cortex (Ctx) or tail (floxed 335 bp band only). Genomic DNA was isolated from indicated tissues from female and male Kiss-PTEN KO (Kiss-Cre^+^/PTEN^flx/flx^) mice. (**B**) Representative images of double-immunofluorescent labeling of PTEN and GFP in Kiss-PTEN KO/R26-YFP mice show that the loss of PTEN expression is restricted to Kiss-YFP+ neurons. White arrows indicate double-labeled kisspeptin neurons. Scale bar = 10 μm. 3V, third ventricle. (**C**) The percentage of Kiss-YFP+ cells co-expressing PTEN in both the anteroventral periventricular nucleus (AVPV) and arcuate nucleus (ARC) is lower in Kiss-PTENKO/R26-YFP animals (*n* = 2–3/genotype and sex). Values are means ± SEM. * *p* < 0.05, ** *p* < 0.01.

Using PCR, we first confirmed that Cre-mediated deletion of the *Pten* sequence flanked by LoxP sites was specific to tissues expressing *Kiss1* in adult mice of both sexes, including the mediobasal hypothalamus (MBH), the liver, and the gonads, while the deletion allele was not detected in tissues that lack kisspeptin expression, namely the neocortex and the tail (Figure 1A). To further validate the loss of PTEN expression in kisspeptin neurons, we performed double immunofluorescence analysis in Kiss-PTEN KO and WT mice carrying a *Rosa26-eYFP* reporter (Kiss-PTEN KO/R26-YFP and Kiss/R26-YFP, respectively; Figure 1B). We found that PTEN was co-expressed in ~30-40% of hypothalamic kisspeptin neurons in Kiss/R26-YFP (WT) males and females. In contrast, the percentage of PTEN co-labeled kisspeptin neurons was much lower in Kiss-PTEN KO/R26-YFP (Figure 1C). In the AVPV, we found that the number of kisspeptin/PTEN co-labeled cells was reduced from 30.8 ± 7.5% in WT to 18.4 ± 1.0% in Kiss-PTEN KO males and from 48 ± 9.5% to 10 ± 1.9% in Kiss-PTEN KO females (Figure 1C). In the ARC, Kiss-PTEN KO mice exhibited an even greater reduction in the percentage of kisspeptin cells co-expressing PTEN (males, Kiss-PTEN KO: 12.4 ± 2.7% vs. WT: 32.7 ± 2.8%; females, Kiss-PTEN KO 5.4 ± 0.5% vs. WT: 32.8 ± 4.0%, Figure 1C).

### 2.2. Deletion of Phosphatase and Tensin Homolog (PTEN) in Kisspeptin Cells Results in Subfertility in Female Mice

We did not observe a genotype effect on adult body weight (Table 1) and the animals displayed general good health throughout the study. Hypothalamic kisspeptin neurons are important for pubertal development in mammals [5,6], therefore we recorded puberty onset in WT and KO littermates using external markers such as preputial separation in males and day of vaginal opening and first day of estrus in females. In males, we did not find a genotype effect regarding the day of full preputial separation (Table 1). Similarly, in females, we did not observe a genotype effect on the age of vaginal opening or day of first estrus (Table 1).

Fertility assessment in males did not reveal a genotype effect, with mice of both genotypes equally successful in siring pups (Table 1). In adult males, no significant differences were observed in wet testicular weight between genotypes (Table 1). Histological examination of cross-sectioned testicular tissue showed normal seminiferous tubules with all types of spermatogonic cells and spermatozoa present in both WT and Kiss-PTEN KO adult males (Supplementary Figure S1A).

Adult females of both genotypes showed normal estrous cyclicity when compared to WT littermates, however, Kiss-PTEN KO females had a lower percentage of successful litter-bearing pregnancies (Table 1). We did not observe a difference in the number of pups produced by Kiss-PTEN KO and WT females that were able to get pregnant (Table 1). Histological examination of the ovaries did not reveal genotype differences in the number of corpora lutea (CL) per ovary in adult females (Supplementary Figure S1B, Table S1).

### 2.3. Gonadectomy-Induced Luteinizing Hormone (LH) Release Is Attenuated in Kiss-PTEN KO Mice

**Figure 2:**
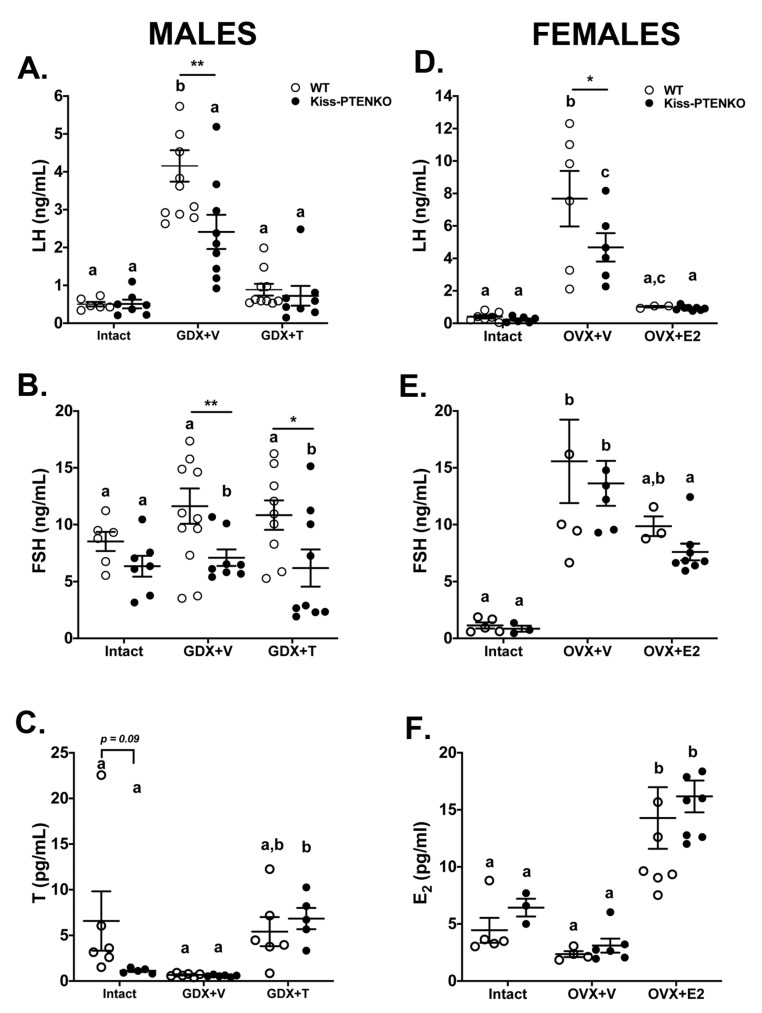
Gonadotropin and sex hormone concentrations from intact, gonadectomized (GDX) and GDX plus steroid hormone-treated WT and Kiss-PTEN KO mice. (**A**) Plasma luteinizing hormone (LH), (**B**) follicle stimulating hormone (FSH), and (**C**) testosterone (T) levels from gonad-intact or GDX adult WT and Kiss-PTEN KO males treated with vehicle (GDX+V) or with T-containing capsules (GDX+T) for 10 days (*n* = 5–10 mice/treatment/genotype). (**D**) Plasma LH, (**E**) FSH, and (**F**) estradiol (E_2_) levels from ovary-intact diestrus or ovariectomized (OVX) adult WT and Kiss-PTEN KO females treated with vehicle (OVX+V), or with E_2_ capsules (OVX+E2) for 10 days (*n* = 5–10 mice/treatment/genotype). All data are means ± SEM. Different letters denote significant differences (two-way ANOVA, Holm Šidák post-hoc). * *p* < 0.05, ** *p* < 0.01, denotes significant differences between genotypes.

We next assessed whether PTEN deletion in kisspeptin cells affected the response of circulating gonadotropin levels following gonadectomy and under E_2_ or testosterone (T) negative feedback. In males, we detected a main effect of genotype on LH levels (two-way ANOVA, effect of genotype, *p* < 0.05). In addition, we found significant differences in plasma LH levels depending on treatment and genotype (*p* < 0.05), with gonadectomized plus vehicle (GDX+V) Kiss-PTENKO males showing a reduced LH response after GDX compared to GDX+V WT males (Holm Šidák post-hoc test, *p* < 0.01; Figure 2A). A main treatment effect was observed in both genotypes (*p* < 0.001) as GDX+V treated mice had increased, while GDX+T mice had decreased plasma LH concentrations (Figure 2A). An overall genotype effect was observed on FSH levels as well (two-way ANOVA, effect of genotype, *p* < 0.05) with post hoc tests showing FSH levels in GDX+V and GDX+T Kiss-PTEN KO males being significantly lower than those in GDX+V and GDX+T WT males, respectively (Holm Šidák post-hoc test *p* < 0.01 GDX+V, *p* < 0.05, GDX+T, Figure 2B). Plasma T levels were similar between gonad-intact mice of both genotypes, Kiss-PTEN KO males, however, showed a trend towards lower T (*p* = 0.089). As expected, we observed a significant treatment effect, as hormone replacement using T capsules successfully restores physiological T levels (two-way ANOVA, effect of treatment, *p* < 0.01, Figure 2C).

A genotype effect (two-way ANOVA, *p* < 0.05) and a genotype and treatment interaction (two-way ANOVA, *p* < 0.01) on LH levels were also obvious in females (Figure 2D). Kiss-PTEN KO females had a significantly reduced LH response to ovariectomy (OVX) compared to OVX+V WT females (Holm Šidák post-hoc, *p* < 0.001, Figure 2D). We also found a treatment effect, with OVX increasing plasma LH levels while E_2_ replacement decreased LH levels in females of both genotypes (two-way ANOVA, effect of treatment, *p* <0.001, Figure 2D). There was a strong treatment effect on plasma FSH levels in females as well (two-way ANOVA, *p* < 0.001; Figure 2E), however no genotype effect was observed. Plasma E_2_ concentrations in ovary-intact females at diestrus were not significantly different between genotypes, however a treatment effect was observed (two-way ANOVA, effect of treatment, *p* < 0.001) as both OVX+E_2_ WT and OVX+E_2_ Kiss-PTEN KO females responded to hormone replacement with increased E_2_ levels (Figure 2F).

### 2.4. Kisspeptin-Cell Specific Deletion of PTEN Alters the Number of Anteroventral Periventricular Nucleus (AVPV) Kisspeptin-Immunoreactive Neurons

**Figure 3:**
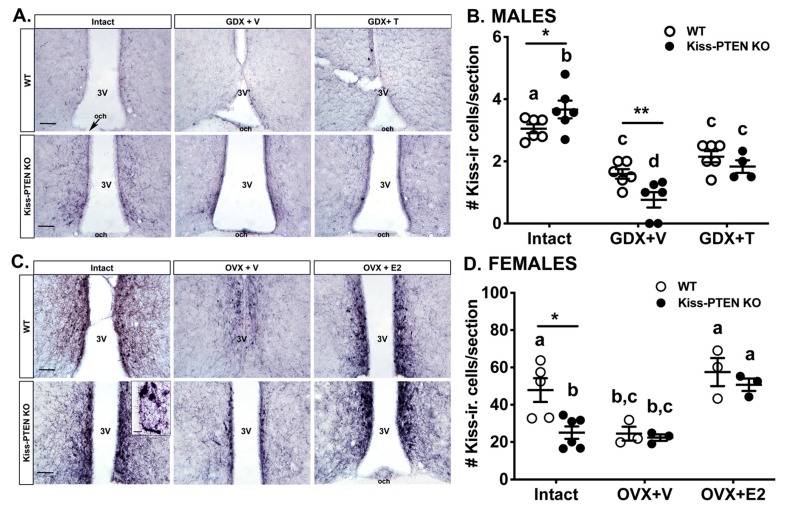
Altered kisspeptin neuron-immunoreactivity (ir) in the AVPV of Kiss-PTEN KO mice. (**A**) Representative images of immunostaining against kisspeptin in the AVPV of gonad-intact, or gonadectomized adult WT and Kiss-PTEN KO males treated with vehicle (GDX+V) or with T-containing capsules (GDX+T) for 10 days. Scale bar = 100 μm. 3V, third ventricle, och, optic chiasm. (**B**) Quantification of kisspeptin-ir cells from each group in (**A**). Values are means ± SEM, (*n* = 4–6/treatment/genotype). Different letters denote significant differences (two-way ANOVA, Holm Šidák post-hoc). *p* < 0.05, ** *p* < 0.01, denotes significant differences between genotypes. (**C**) Representative images of AVPV kisspeptin-ir from gonad-intact, or OVX adult WT and Kiss-PTEN KO females treated with vehicle (OVX+V) or with E_2_-containing capsules (OVX+E2) for 10 days. Scale bar = 100 μm. 3V, third ventricle, och, optic chiasm. (**D**) Quantification of kisspeptin-ir cells from each group in (**C**). Values are means ± SEM (*n* = 3–6/treatment/genotype). Different letters denote significant differences (two-way ANOVA, Holm Šidák post-hoc). * *p* < 0.05, denotes significant differences between genotypes.

PTEN signaling regulates neuronal cell proliferation and protein synthesis [14], therefore, we performed nickel-enhanced 3,3′-Diaminobenzidine (DAB) immunohistochemistry (IHC) to examine the effects of PTEN ablation on kisspeptin-immunoreactivity (ir) in intact and GDX adult mice with and without sex hormone replacement. We found that kisspeptin-ir in the AVPV of Kiss-PTEN KO males showed a stronger neuropil staining intensity when compared to controls and observed more kisspeptin-ir fibers in all treatment conditions (Figure 3A). While we did not find an effect of genotype on AVPV kisspeptin-ir cell number, we did however observe an effect of treatment (two-way ANOVA, *p* < 0.05) and an interaction between genotype and treatment (two-way ANOVA, *p* < 0.5). *Post-hoc* analysis revealed kisspeptin-ir cell numbers to be higher in gonad-intact Kiss-PTEN KO mice compared to intact WT controls (Holm Šidák post-hoc test, *p* < 0.05, Figure 3B). In contrast, within the GDX+V group, the number of kisspeptin-ir cells was lower in AVPV Kiss-PTEN KO mice compared to WT (*p* < 0.01, Figure 3B).

As in males, ablation of PTEN in kisspeptin neurons resulted in intense AVPV kisspeptin-ir compared to WT females across treatment groups (Figure 3C). In addition, we found larger cell bodies clustered together along the third ventricle in the AVPV of Kiss-PTEN KO females (Figure 3C, insert), contrasting with the more separate kisspeptin-ir cell bodies in the AVPV of WT females. A trend towards a genotype effect on AVPV kisspeptin-ir cell number was observed (two-way ANOVA, *p* = 0.07). However, a significant treatment effect was evident, with OVX reducing the number of AVPV kisspeptin-ir cells and E_2_ replacement increasing it in both genotypes (Figure 3D). *Post-hoc* multiple comparison analysis revealed that within the intact group, Kiss-PTEN KO females had reduced AVPV kisspeptin-ir cell numbers when compared to intact WT females (*p* <0.05, Figure 3D). Moreover, no significant differences were detected between intact and OVX + V KO females, whereas in WT females, the number of kisspeptin-ir cells were significantly lower in OVX + V compared to both the intact and the E_2_-replaced group (*p* < 0.05, Figure 3D).

**Figure 4:**
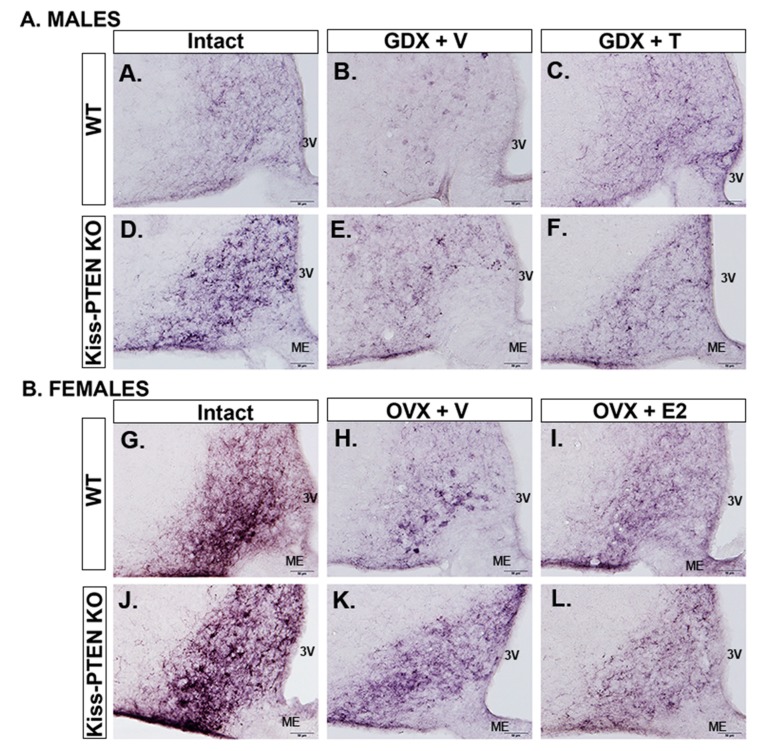
ARC kisspeptin-ir is not affected by gonadectomy in Kiss-PTEN KO males and females. Representative images showing kisspeptin-ir fiber plexus in the ARC of WT and Kiss-PTEN KO males (**A**–**F**) and females (**G**–**L**). Animals were either gonad-intact, gonadectomized treated with vehicle (GDX+V, males; OVX+V, females), or with steroid hormone replacement (GDX+T, males; OVX+E2, females). Scale bar = 100 μm. 3V, third ventricle, ME, median eminence.

We also examined the changes in ARC kisspeptin immunostaining patterns in response to gonadectomy and steroid hormone replacement in both genotypes and sexes (Figure 4). As previously shown [16], we observed a dense kisspeptin fiber plexus in the ARC of adult WT mice of both sexes, with a denser fiber immunoreactivity in diestrus females compared to intact males, and a lack of identified kisspeptin cell bodies (Figure 4A,G). Qualitative assessment of ARC kisspeptin staining pattern suggested that in WT males, GDX notably reduced kisspeptin fiber-ir, allowing the visualization of kisspeptin-ir cell bodies (Figure 4C). In contrast, the reduction of kisspeptin fiber-ir after GDX was not as pronounced in Kiss-PTEN KO males (Figure 4D). Testosterone replacement in males of both genotypes resulted in ARC kisspeptin immunostaining patterns resembling the gonad-intact state (Figure 4E,F). The effects of PTEN ablation on ARC kisspeptin fiber-ir were more dramatic in females (Figure 4G–L). Female WT mice that underwent OVX showed a reduction in ARC kisspeptin fiber immunostaining and darkly stained cell bodies were revealed (Figure 4I). In contrast, the ARC kisspeptin staining pattern in OVX+V Kiss-PTEN KO females was qualitatively similar to ovary-intact Kiss-PTEN KO females, with individual kisspeptin cell bodies not discernable among the dense fiber plexus (Figure 4J). Lastly, E_2_ replacement in females (OVX+E_2_) resulted in ARC kisspeptin fiber-ir similar as in intact females (Figure 4K,L).

### 2.5. Sex- and Nucleus-Specific Effects on Kisspeptin Neuron Numbers and Cell Size in Kiss-PTEN KO Mice

**Figure 5:**
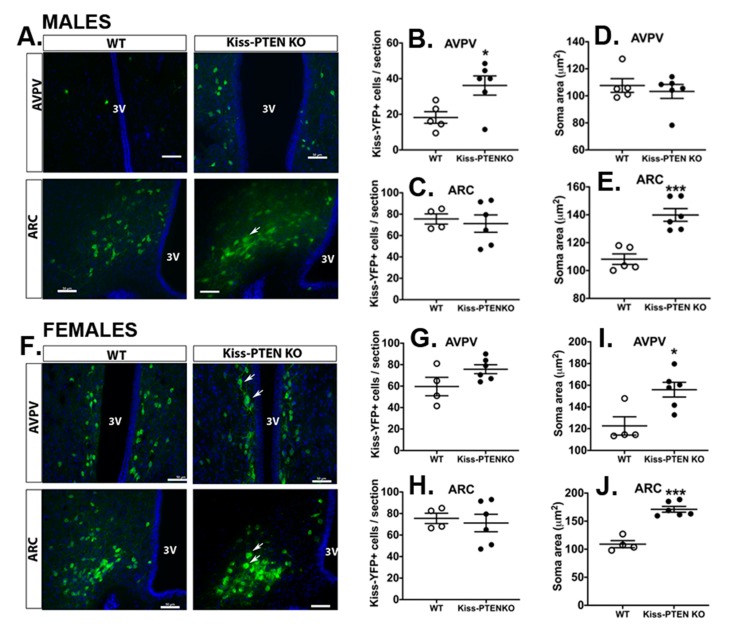
Sex and region-specific kisspeptin neuron hypertrophy in Kiss-PTEN KO mice. (**A**) Representative immunofluorescent images show higher number of Kiss-GFP+ neurons in the AVPV as well as increased fiber staining and larger cell bodies in the ARC (white arrow) of Kiss-PTEN KO males. 3V, third ventricle. Scale bars, 50 μm. (**B**,**C**) Quantification of Kiss-GFP+ cells in the AVPV and ARC of males. (**D**,**E**) Kisspeptin cell soma area measurement in the AVPV and ARC areas in males (*n* = 5–6 /genotype). (**F**) Representative immunofluorescent images show larger Kiss-GFP+ neurons in the AVPV and ARC of Kiss-PTEN KO females (white arrow). 3V, third ventricle. Scale bars, 50 μm. (**G**,**H**) Quantification of Kiss-GFP+ cells in the AVPV and ARC of WT and Kiss-PTEN KO females. (**I**,**J**) Kisspeptin cell soma area measurement in both AVPV and ARC (*n* = 4–6/genotype). Values are means ± SEM. Unpaired t-test, * *p* < 0.05, *** *p* < 0.001.

While utilizing DAB staining to assess the number of kisspeptin-ir cells in the AVPV, we noticed that the kisspeptin-ir fibers surrounding the cell bodies were intensely stained and dense in the Kiss-PTEN KO animals, particularly in females, impeding an accurate assessment of kisspeptin positive cell numbers. Furthermore, the intense kisspeptin-ir fiber plexus in the ARC precluded semi-quantitation of the number of kisspeptin-expressing neurons in both males and females. Therefore, to better assess the number and morphology/size of hypothalamic kisspeptin neurons in WT and KO animals, we generated a Kiss-PTEN KO/R26-YFP reporter mouse line to visualize kisspeptin neurons by immunofluorescence using antibodies against GFP (Figure 5).

Consistent with our DAB immunostaining results, Kiss-PTEN KO/R26-YFP intact males had more Kiss-GFP+ neurons in the AVPV compared to WT (Kiss/R26-YFP) intact males (*p* < 0.01; Figure 5B). In contrast, we did not detect differences in the numbers of ARC Kiss-GFP+ neurons between WT and Kiss-PTEN KO/R26-YFP males (Figure 5C). In agreement with the DAB staining results, we observed an apparent increase in fiber density in the ARC of Kiss-PTEN KO/R26-YFP males (Figure 5A, lower panels).

In females, Kiss-GFP+ cell counts in the AVPV and the ARC were not significantly different between WT and Kiss-PTEN KO/R26-YFP mice (Figure 5G,H). As in males, fiber-ir appeared to be increased in the ARC of Kiss-PTEN KO/R26-YFP females when compared to WT mice (Figure 5F, lower panels).

PTEN deletion also had sex- and region-specific effects on kisspeptin neuronal size. Specifically, kisspeptin neurons in Kiss-PTEN KO/R26-YFP females had a larger soma area in both the AVPV (*p* < 0.05, Figure 5I) and the ARC (*p* < 0.001, Figure 5J) when compared to WT controls. Interestingly, kisspeptin neuron hypertrophy in Kiss-PTEN KO/R26-YFP males was restricted to the ARC (*p* < 0.001, Figure 5E).

### 2.6. Hyperactivation of mTOR Signaling in Kisspeptin Neurons of Kiss-PTEN Knockout Females but not Males

**Figure 6:**
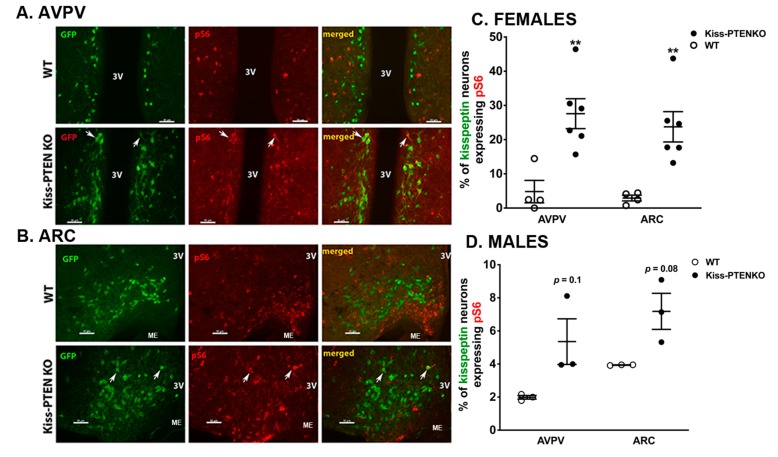
Increased downstream mTOR signaling in female Kiss-PTEN KO AVPV and ARC kisspeptin neurons. Representative fluorescent images of Kiss-GFP+ neurons (green), pS6 (red), a marker of mTOR activity, and co-localization (merged, *white arrow*) in the AVPV (**A**) and ARC (**B**) of WT and Kiss-PTEN KO/R26-YFP females. Scale bar = 50 μm. 3V, third ventricle, ME, median eminence. (**C**) Percentage of Kiss-GFP+ neurons that co-express pS6 in the female AVPV and ARC (*n* = 4–6/genotype). (**D**) Percentage of Kiss-GFP+ neurons that co-express pS6 in the male AVPV and ARC (*n* = 3/genotype). Unpaired t-test, ** *p* < 0.01.

PTEN is the principal negative regulator of the PI3K signaling pathway and associated downstream effector signaling molecules such as mTOR. The effects of neuronal *Pten* deletion have been associated with an increase in mTOR activity [14]. Thus, we performed double-immunofluorescent labeling of Kiss-GFP+ neurons with phospho-S6 (pS6), a downstream effector of mTOR, to determine whether deletion of *Pten* resulted in mTOR hyperactivity in hypothalamic kisspeptin neurons. Compared to Kiss/R26-YFP (WT) females, the percentage of Kiss-GFP+ neurons co-labeled with pS6 was significantly higher in both the AVPV (Figure 6A) and ARC (Figure 6B) of Kiss-PTEN KO/R26-YFP females (*p* < 0.01, Figure 6C). However, although we observed an increase in pS6 co-labeling of kisspeptin neurons in the AVPV and ARC of Kiss-PTEN KO/R26-YFP males, this increase did not reach statistical significance (Figure 6D).

### 2.7. Maintenance of LH Levels and Hypothalamic Kisspeptin Protein Expression after Fasting in Kiss-PTEN KO Females

States of negative energy balance, such as nutritional deprivation, results in suppression of gonadotropin release in females and are associated with a decrease in hypothalamic kisspeptin expression [3]. mTOR is both a cellular nutrient sensor and an important central regulator of energy homeostasis [17,18]. Therefore, we next investigated whether the increase mTOR activity in kisspeptin neurons of Kiss-PTEN KO females was associated with changes in the gonadotropin response to a 48 h fast.

**Figure 7:**
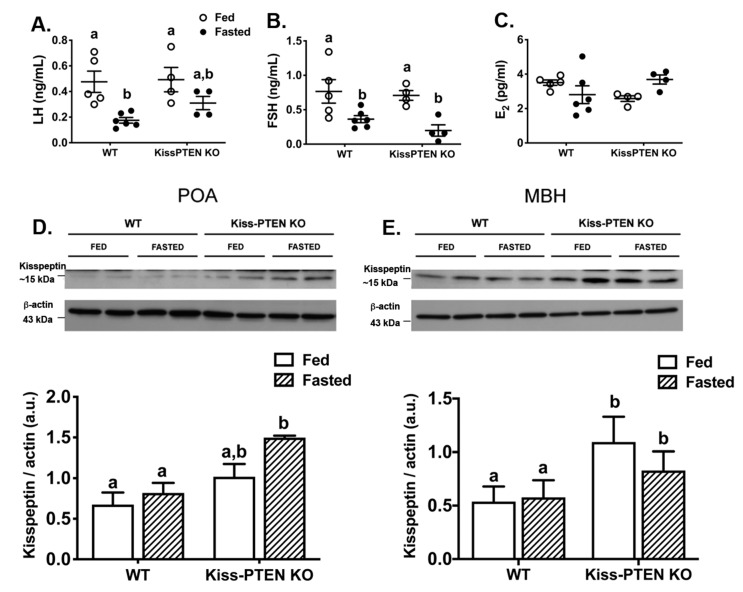
Kiss-PTEN KO females maintain basal LH after fasting and express more hypothalamic kisspeptin protein compared to fasted WT females. (**A**) Terminal plasma LH, (**B**) FSH, and (**C**) E_2_ levels in WT and Kiss-PTEN KO females that were either left fed or fasted for 48 h. All females were sacrificed at diestrus (*n* = 4–6 mice/diet/genotype). (**D**) Immunoblots performed for kisspeptin in the preoptic area (POA) and (**E**) the MBH; full blots are shown in Supplementary Figure S2. Densitometry analysis of kisspeptin levels relative to β-actin are also shown (*n* = 4 mice/diet/genotype). Values are means ± SEM. Different letters denote significant differences (two-way ANOVA, Holm Šidák post-hoc). POA, preoptic area, MBH, mediobasal hypothalamus; a.u., arbitrary units.

Females subjected to a 48-h fast weighted significantly less than fed females and no effect of genotype on weight loss was observed (2-way ANOVA; p > 0.05). Compared to non-fasted controls, fasted females showed reduced blood glucose levels regardless of genotype (in mg/dL: WT fed 115 ± 2 vs. WT fasted 75 ± 7; KO fed 130 ± 5 vs. KO fasted 65 ± 9). As expected, fasting significantly decreased plasma LH levels in WT females (*p* < 0.01, Figure 7A); in contrast, plasma LH levels in fasted Kiss-PTEN KO females were not significantly different from fed controls of both genotypes. On the other hand, plasma FSH levels were significantly lower in fasted females of both genotypes (*p* < 0.05, Figure 7B). Estradiol levels however, were not significantly different between fed and fasted WT females or Kiss-PTEN KO females (Figure 7C). Interestingly, a significant diet and genotype interaction was observed (two-way ANOVA, *p* < 0.05) reflected in slightly elevated plasma E_2_ levels in fasted Kiss-PTEN KO females (Figure 7C).

We next investigated whether the effect that PTEN deletion has in maintaining LH levels in fasted Kiss-PTEN KO females is associated with differences in hypothalamic kisspeptin protein levels. Western blot analysis showed that the kisspeptin peptide content in the preoptic area (POA) was higher overall in Kiss-PTEN KO females (two-way ANOVA, genotype effect, *p* < 0.01, Figure 7D). In addition, an effect of diet was observed (two-way ANOVA, *p* < 0.05); specifically, fasted Kiss-PTEN KO females had an increased kisspeptin protein content compared to fed and fasted WT females (Tukey’s multiple comparisons, *p* < 0.01 and *p* < 0.05, respectively). Similarly, in the MBH (Figure 7E), we also observed a genotype effect (two-way ANOVA, *p* < 0.05, Figure 7E), with Kiss-PTEN KO fed and fasted females expressing more kisspeptin protein than WT females. However, diet did not affect MBH protein levels in either genotype.

## 3. Discussion

PTEN switches off the PI3K pathway by directly dephosphorylating the second messenger phosphatidylinositol (3,4,5)-triphosphate (PIP3) to phosphatidylinositol (4,5)-bisphosphate (PIP2), resulting in the inhibition of leptin and insulin signaling [13]. PTEN is also regulated by estrogen, contributing to this hormone’s trophic effects [19,20,21]. Moreover, cell-specific PTEN deletion models suggest that this signaling molecule plays sexually dimorphic functions pertaining to metabolic control [22]. Hypothalamic kisspeptin neurons are important sites for the actions of sex hormones and metabolic signals which indirectly regulate GnRH neuronal function [2]. Because the intracellular signaling of these peripherally-derived hormones converge on the PI3K pathway, we deleted *Pten* in kisspeptin-expressing cells, which is expected to result in elevated PIP3 levels and enhanced PI3K activity [22].

Kisspeptin neurons in the AVPV are sexually dimorphic, with adult females having over 10-fold more kisspeptin-ir cell bodies than males [23]. Studies in animal models of low reproductive hormone levels such as the hypogonadal (*hpg*) mice, which are GnRH-deficient, and in aromatase knockout (*ArKO*) mice, which cannot convert T to E_2_ suggest that both organizational as well as acute activational actions of E_2_ play a key role in sexually dimorphic AVPV kisspeptin expression. For example, the data obtained on the maturation of kisspeptin neurons in these models suggest that perinatal E_2_ exposure is required for the full development of the AVPV kisspeptin population, as the sexual dimorphism normally observed in AVPV kisspeptin neurons is no longer evident and treatment of adult females cannot restore kisspeptin-ir cell numbers [24,25,26]. Similar to what has been reported in *hpg* and *ArKO* mice, we observed a reduction in AVPV kisspeptin-ir cell number in Kiss-PTEN KO females, whereas adult Kiss-PTEN KO males showed an increase in the number of AVPV kisspeptin-ir cells, a phenotype resembling that of *hpg* males [25]. However, we found that regardless of genotype, replacement with E_2_ and T restored the number of kisspeptin-ir cell bodies in the AVPV to WT levels, an effect not observed in *hpg* or *ArKO* models [24,25]. Hence, our data suggest that the AVPV kisspeptin-ir cell number phenotype observed in Kiss-PTEN KO animals is not developmentally preprogrammed. Alternatively, the regulation of kisspeptin neurons by intracellular signaling molecules such as PTEN may involve sex steroid-independent mechanisms, such as those utilized by growth factors like Insulin-like growth factor 1 [27,28]) and insulin [29,30].

In addition to the effects on kisspeptin-ir cell numbers, deletion of PTEN markedly increased ir density of the fiber staining for kisspeptin, particularly in the ARC. Activation of PI3K and the subsequent generation of PIP3 result in the recruitment of AKT1 to the plasma membrane. AKT1 activates mTOR, which in turn initiates protein translation via downstream effectors S6 kinase 1 (S6K1) and eukaryotic initiation factor 4E-binding protein 1 (EIF4EBP1, also known as 4E-BP1) [18]. We found that the hyperactivation of this pathway after PTEN deletion was sex-specific, with a significant increase in the number of Kiss-GFP+ cells co-expressing pS6 only in females. Estrogen has been shown to stimulate mTOR-mediated protein synthesis via the activation of extracellular signal-regulated kinase (ERK) and Akt pathways and via PTEN degradation [20]. Moreover, a recent study showed that OVX down-regulates mTOR signaling in the ARC, an effect that was reversed by either E_2_ replacement or central administration of ERα agonists [31]. Hence, the increase in mTOR activity observed in the hypothalamus of Kiss-PTEN KO females may be due to the actions of E_2_ on this pathway. On the other hand, the increase in kisspeptin immunoreactivity observed in Kiss-PTEN KO males might be attributed to PI3K-AKT pathway-independent functions of PTEN. For example, an alternative pathway used by PTEN to inhibit protein synthesis involves its interaction with and activation of the RNA-dependent protein kinase (PKR) which in turn phosphorylates the alpha subunit of eukaryotic translation initiation factor 2 (eIF2α), inhibiting translation [32].

The mTOR signaling pathway is divided into two arms, namely mTOR complex 1 (mTORC1), which requires the activation of the Rapamycin-sensitive adapter protein Raptor, and the mTOR complex 2 (mTORC2), which requires the Rapamycin-insensitive adaptor Rictor [14,18]. In the CNS, activation of mTORC2 signaling has been implicated in the regulation of soma size, cytoskeletal organization and dendritic growth [14,33,34]. For example, Rictor-deficient neurons are smaller and have altered neurite organization [33]. mTORC2 downstream targets include Akt and the serum- and glucocorticoid-related kinase (SGK), which are part of growth factor signaling pathways [35]. Therefore, the increase in kisspeptin soma size observed in the AVPV of Kiss-PTEN KO females and in the ARC of both sexes might be mediated by the mTORC2 branch of the mTOR signaling pathway.

Despite significant sex- and brain-region specific effects on kisspeptin-ir pattern, basal LH and FSH levels were similar between WT and Kiss-PTEN KO animals. Moreover, regardless of genotype, steroid hormone replacement in gonadectomized animals reduced LH levels. However, the increase in LH after GDX was lower in Kiss-PTEN KO mice compared to GDX WT counterparts. In addition, GDX in kisspeptin-PTEN KO mice failed to decrease ARC kisspeptin-ir fiber density which would have resulted in the visualization of kisspeptin-ir cell bodies [25]. ARC kisspeptin neurons co-express two other neuropeptides, neurokinin B (NKB) and dynorphin A, and it has been proposed that these neurons, referred to as KNDy neurons, form a network that acts in a paracrine fashion to control peptide output [4,36]. In fact, ARC KNDy neurons influence LH release indirectly through their projections to GnRH nerve terminals that run through the ARC to the median eminence (ME), participating in the steroid regulation of GnRH/LH pulse frequency [23,37,38]. KNDy neurons also express ERα and are sensitive to E_2_ suppression of LH [9,39,40]. Our results agree with other studies showing that ARC kisspeptin neurons are not essential mediators of E_2_ negative feedback. For example, mice with a kisspeptin cell-specific ablation of ERα respond normally to E_2_ negative feedback on LH, yet these mice also exhibited a blunted LH increase following gonadectomy [9]. Similarly, a reduced LH increase after GDX was reported in a study where ERα was selectively ablated in the ARC of female mice [40], as well as in a study where ablation of KNDy neurons in the ARC of female rats prevented the rise in serum LH after OVX and attenuated the rise in serum FSH levels [41]. It remains unclear why disruption of ARC KNDy neurons results in a diminished LH response to GDX. It has been suggested that OVX results in a more efficient electrochemical coupling of kisspeptin release from kisspeptin nerve terminals. However, in a study in which PTEN was deleted in dopamine (DA) neurons, no alterations in basal DA extracellular levels or evoked DA release were observed, despite a significant increase in total tissue levels [42]. On the other hand, others have shown that PTEN regulates neuronal firing activity through modulation of ATP-sensitive potassium (K_ATP_) channels, which are themselves targets of steroid hormone regulation [43]. Hence, it is possible that deletion of PTEN resulted in K_ATP_ channel activation and hyperpolarization of a subset of ARC kisspeptin neurons in the gonadectomized group, altering the normal increase in GnRH/LH release.

PTEN’s role in opposing leptin- or insulin-stimulated PI3K signaling also contributes to its ability to centrally control energy homeostasis. For instance, females but not males, carrying a deletion of *Pten* in POMC-expressing neurons exhibit hyperphagia and elevated body weights after a high fat diet [22]. Kisspeptin neurons are also targets of insulin and leptin signaling, thereby mediating the impact of nutritional status on GnRH release [30,44]. The rapamycin-sensitive mTORC1, which is a downstream target of PI3K/Akt signaling, has been linked to the activation of the HPG axis at puberty and to the regulation of *Kiss1* expression and LH levels by metabolic status [45,46]. For example, chronic administration of L-leucine partially rescues the suppression of LH levels induced by persistent chronic food restriction of peripubertal female rats [45]. Conversely, pharmacological inhibition of mTOR by central administration of rapamycin decreases LH levels and hypothalamic *Kiss1* mRNA expression in pubertal female rats, an effect dependent on the presence of estrogen. Inactivation of mTOR was also shown to diminish the positive effect of leptin on puberty onset in food-restricted females. In our model, where PTEN deletion elicited a significant increase in pS6 in kisspeptin neurons of females, we hypothesized that constitutive activation of mTOR would modulate both the LH response and kisspeptin protein expression in females after a 48-h fast. As expected, a decrease in circulating LH after fasting was observed in WT females; in contrast, Kiss-PTEN KO females were able to maintain basal LH levels after fasting suggesting that kisspeptin neurons may act as a specific neuroanatomical site where active mTOR (induced by the loss of PTEN) can rescue deficient LH levels under negative energy balance. We also observed that regardless of genotype, fasting did not decrease kisspeptin protein levels in the POA or the MBH. The lack of effect on kisspeptin protein expression by fasting might be due to the high sensitivity this neuropeptide system has to E_2_ levels. For example, a 48 h fast reduces AVPV *Kiss1* mRNA expression and LH levels in OVX adult rats receiving E_2_ replacement but not in OVX animals devoid of E_2_ [47]. Furthermore, in this study, regardless of E_2_ treatment, fasting did not change ARC *Kiss1* mRNA expression. Our females were sacrificed in the morning of diestrus, when E_2_ levels are low, hence AVPV kisspeptin protein levels might have been already too low to detect any effect by fasting. In contrast, Kiss-PTEN KO females maintained hypothalamic kisspeptin protein levels that were higher than either fed or fasted WT females. Altogether, these data suggest that PTEN signaling plays a role in maintaining LH levels in females after fasting, possibly by regulating hypothalamic kisspeptin protein content.

Compared to WT, mating success was lower in Kiss-PTEN KO females. The mechanism underlying the decrease in fertility in Kiss-PTEN KO females is unclear as they showed normal cyclicity and normal CL numbers. Even though we focused on hypothalamic kisspeptin neurons since they are a major driver of GnRH/LH release, we cannot rule out that *Pten* deletion in extra-hypothalamic kisspeptin cells contributed to this reproductive deficit in females. For example, in the mouse uterus, kisspeptin as well as PTEN signaling are important in embryo implantation [48,49]. However, while PTEN signaling has been linked to the decidualization process and successful trophoblast invasion [49], the kisspeptin system in the uterus specifically controls embryo attachment through its regulation of Leukemia inhibitory factor (Lif) expression [48]. Therefore, future studies will determine whether the subfertility observed in Kiss-PTEN KO females is of central or peripheral origin.

In conclusion, our data demonstrate that PTEN functions as a modulatory signaling mechanism whereby sex steroids and metabolic cues are integrated by kisspeptin neurons, establishing PTEN as an important contributor in the regulation of kisspeptin protein expression as well as in its function as LH secretagogue.

## 4. Materials and Methods

### 4.1. Animals

Animals were housed at Stony Brook University, Division of Laboratory Animal Resources under a 12:12 light-dark cycle (lights ON at 7:00 am) and had access to water and rodent chow *ad libitum*. All procedures were approved by the Institutional Animal Care and Use Committee at Stony Brook University Medical Center in accordance with the NIH Guide for the Care and Use of Laboratory Animals (IRB # 214028-26).

### 4.2. Generation of Kiss-PTEN KO Mice

All animals used were bred on a C57BL/6J genetic background. To specifically ablate *Pten* in kisspeptin neurons, mice with exons 4 and 5 of *Pten* flanked by loxP sites (PTEN^flx/flx^) [22,50,51,52,53] were mated with mice expressing Cre recombinase under the control of the endogenous *Kiss1* promoter (Kiss-IRES-Cre mice) [8,39,54]. Offspring generated in the first breeding was then intercrossed to generate Kiss-Cre^+^/PTEN^flx/flx^ (Kiss-PTEN KO) and their Kiss-Cre^-^/PTEN^flx/flx^ and Kiss-Cre^-^/PTEN^flx/wt^ (wild-type; WT) littermates. Where indicated, mice were also crossed with R26-stop-EYFP knock-in mice (Jackson Laboratories, Bar Harbor, ME, USA, stock #006148), which served as a reporter to monitor Cre-mediated recombination. Animals were genotyped for the presence of Cre and floxed PTEN through polymerase chain reaction (PCR) of isolated genomic tail DNA as described previously [50,53]. We also used PCR to screen for germline recombination and deletion of *Pten* using DNA from the tail and other tissues devoid of kisspeptin cells by PCR as described previously [50,52,53]. Mice with non-specific *Pten* deletion were excluded from these studies.

### 4.3. Pubertal Onset and Estrous Cyclicity Assessment

Vaginal opening in females and balanopreputial separation in males was assessed daily starting at weaning age (i.e., postnatal day 21). To determine the age of first estrus, vaginal cells were collected by lavage using 0.9% saline starting at the day of vaginal opening and continuing each day thereafter. To examine the possible effects of genotype on estrous cyclicity, vaginal lavage was collected from young adult females (2 months old) and vaginal cytology was examined under a microscope daily (9:00–11:00 am) for 15 days. A normal estrous cycle was defined as exhibiting predominantly leukocytic vaginal epithelial cells for 2 days followed by 1 day of nucleated cells and 1–2 days of cornified vaginal cells.

### 4.4. Fertility Studies

WT and Kiss-PTEN KO females were paired with a proven fertile WT male for 7 days and females were checked for copulatory plugs daily. Following the 7-day mating period, females were housed separately for 21 days. Four rounds of pairing were evaluated, in which WT stud males were alternated between pairings with WT and Kiss-PTEN KO females. Gestation time and litter size were recorded. Male fertility was assessed by pairing WT and Kiss-PTEN KO males with proven fertile WT females for 7 days, while verifying the presence of copulatory plugs. After 7 days, females that were plugged were separated and observed for pregnancy.

### 4.5. Gonad Histology

Ovaries were freshly collected from mice and fixed immediately in 4% paraformaldehyde. Wet testicular weight was determined from freshly dissected young adult male mice followed by fixation in Bouin’s reagent (Sigma-Aldrich, St. Louis, MO, USA) at room temperature. After washing in 70% ethanol, gonads were embedded in paraffin and cut into 5 μm-thick sections (Stony Brook University histology core). Gonadal tissue was then stained with hematoxylin and eosin (H&E) as previously described [54]. The total number of corpora lutea (CL) was counted in a blinded fashion using one ovary per animal. Testes histology was examined and evaluated for the presence and organization of spermatogonic cells.

### 4.6. Gonadectomies and Steroid Hormone Replacement

All gonadectomies (GDX) were done under isoflurane (HenrySchein Animal Health, Dublin, OH, USA) inhalation anesthesia. Adult females of each genotype were randomly assigned to one of three groups: gonadally intact (sham controls), ovariectomy (OVX) + vehicle (V), or OVX+ estradiol (E_2_), (*n* = 5–6/genotype/treatment). Immediately after surgery, animals were implanted with subcutaneous SILASTIC capsules (1.5 cm in length plugged with silicone adhesive on each end to leave 1 cm to fill with treatment; inner diameter 1.47 mm; outer diameter 1.95 mm) containing either sesame oil (OVX+V) or containing 1 mg/mL E_2_ in sesame oil (OVX+E_2_). Ten days after surgeries, animals were killed between 9:00–10:30 am and tissue and brains were collected. Blood plasma was assayed for E_2_, LH, and FSH levels.

Adult males of each genotype were randomly assigned to one of three groups: gonadally intact (sham controls), GDX+V, or GDX+ testosterone (T). Immediately after surgery, animals were implanted with subcutaneous SILASTIC capsules (inner diameter 1.02; outer diameter 2.16 mm) packed with either 5 mg of testosterone (T) (Sigma-Aldrich, St. Louis, MO, USA), or left empty (as vehicle). Animals were killed 10 days after surgery between 9:00–10:30 am and tissue and brains were collected. Plasma was assayed for T, LH, and FSH levels.

### 4.7. Hormone Assays

Plasma luteinizing hormone (LH) and follicle-stimulating hormone (FSH) concentrations were measured using the MILLIPLEX MAP Mouse Pituitary Magnetic Bead Panel MPTMAG-49K (EMD Millipore, Burlington, MA, USA) in a Luminex 200 (Luminex Corp.; Austin, TX, USA). Plasma testosterone (T) levels were measured using an ELISA (R&D Systems, Minneapolis, MN, USA), with a sensitivity of 0.041 ng/mL. Plasma E_2_ was measured using the Mouse/Rat Estradiol ELISA kit #ES180S-100 (Calbiotech, Spring Valley, CA, USA) with a sensitivity of <3 pg/mL.

### 4.8. Perfusion and Immunohistochemistry (IHC) for Kisspeptin

Mice were anesthetized with ketamine (HenrySchein Animal Health, Dublin, OH, USA) and xylazine (AKORN Animal Health, Lake Forest, IL, USA) (100 and 10 mg/kg body weight, respectively) and transcardially perfused with 4% paraformaldehyde in PBS (0.1 M Phosphate buffer saline, pH 7.4). Brains were removed and postfixed for 24 h and then kept in 30% sucrose until saturated. For 3,3′-diaminobenzidine (DAB) immunohistochemistry (IHC) staining, brains were sectioned coronally into sets of 3 at 30-μm thickness. After quenching endogenous peroxidase activity with H_2_O_2_ and washes in TBS, kisspeptin immunoreactivity was detected by incubating sections in TBST (0.3% Triton X-100, 0.25% BSA, and 2% normal goat serum) containing a polyclonal rabbit anti-kisspeptin-10 antibody (AC#566, a gift from Dr. Alain Caraty, Unite de Physiologie de la Reproduction et des Comportements, University of Tours, France [54,55,56], Supplementary Table S1 for antibody information and Supplementary Figure S3 for negative controls omitting primary antibody) for 48 h at 4 °C. Sections were then washed in TBS followed by incubation in biotinylated goat anti-rabbit IgG (1:500; Vector Laboratories, Burlingame, CA, USA) for 1 h, followed by incubation in Vectastain Elite ABC reagent (Vector Laboratories, Burlingame, CA, USA) for 90 min at room temperature. Immunoreactivity was then visualized using the Vectastain nickel-enhanced DAB (NiDAB) Peroxidase substrate kit (Vector Laboratories, Burlingame, CA, USA). Sections were then washed, mounted on slides, and coverslipped using Permount (Thermo Fisher Scientific, Waltham, MA, USA).

### 4.9. KissYFP^+^ Mice and Double Immunofluorescent (IF) Labeling

Knockout and wild-type progeny from Kiss-Cre^+^ PTEN^flx/flx^, or PTEN^flx/wt^ crossed with YFP reporter mice are referred to as Kiss-PTENKO/R26-YFP or Kiss/R26-YFP, respectively. Adult (2–3 months of age) Kiss/R26-YFP mice of both genotypes were transcardially perfused and post-fixed as described above and brains sectioned coronally. Sections were then incubated for 48 h at 4 °C with primary antibodies mouse monoclonal anti-PTEN (1:100, Cell Signaling, Beverly, MA, USA, 9556S, [42,57,58] or rabbit anti-pS6 (1:100, Cell Signaling, Beverly, MA, USA, 2211S [59,60] primary antibodies (Supplementary Table S1 and Supplementary Figure S3 for negative controls omitting primary antibody). This was followed by a 3-hr incubation with Alexa Fluor 594 conjugated anti-mouse or anti-rabbit secondary antibodies (1:500, Life Technologies, Thermo Fisher Scientific, USA) at room temperature. Tissue sections were then incubated in chicken anti-GFP overnight at 4 °C (1:500, Aves Labs Inc., Davis, CA, USA), followed by a 3-h incubation with Alexa Fluor 488 anti-goat (1:500, Life Technologies Life Technologies, Thermo Fisher Scientific, USA) at room temperature. Finally, tissue sections were mounted on slides and coverslipped with DAPI Fluoromount-G (Southern Biotech, Birmingham, AL, USA).

### 4.10. Microscopy for IHC/IF and Image Analysis

All image analysis including neuronal cell counts, single and double labeled cell counts, and soma area measurements were evaluated by at least two individual observers blinded to the genotype at the time of image analysis. NiDAB kisspeptin-immunoreactive (ir) neurons were counted and imaged using a Olympus microscope (model BX 51). Kisspeptin-ir neurons in the AVPV/PeN were quantified as described previously [52]. Kisspeptin-ir neurons of the AVPV were manually counted from 0.62 mm to 0.02 mm anterior to Bregma, and then averaged by the number of sections per animal. Kisspeptin-ir neurons were identified by round, cytoplasmic staining of cell bodies.

For double-IF experiments, Kiss/R26-YFP neurons were imaged using an Axiovert 200M microscope (Zeiss, Germany) and identified by cytoplasmic GFP staining and quantified in the AVPV and ARC using the Image J (NIH) Cell Counter plugin (https://imagej.nih.gov/ij/plugins/cell-counter.html) [39]. Two sections per region per animal were selected for counting neurons and averaged. Kiss/R26-YFP neurons identified with DAPI-labeled nuclei were selected to measure the soma area at 100X oil objective magnification by tracing the somata with the free-hand measurement tool of the Axiovision software (AxioVision 4.8, Zeiss, Germany) [61,62]. For male AVPV Kiss/R26-YFP neurons, a total of 15 neurons (between the two sections used for counting) were selected at random for soma measurements. For female AVPV Kiss/R26-YFP neurons and for ARC Kiss/R26-YFP neurons in both sexes, 40 neurons were randomly selected to measure the soma area. To determine percentage of co-expression in double labeling experiments the number of neurons expressing either PTEN or pS6 was calculated as a fraction of the total number of KissGFP+ neurons counted in two sections of AVPV and two sections of ARC per animal/ per genotype and similarly analyzed in a blinded fashion.

### 4.11. The Effect of 48-h Fasting on LH Levels

Adult WT and Kiss-PTENKO females were either fasted, with free access to water, or left chow-fed for 48 h. After fasting, mice were weighed and fasting blood glucose was measured from the tail using a glucometer to confirm effects of food deprivation. Mice were then killed in the morning at diestrus and blood and tissues were collected. Blood was centrifuged and plasma collected and stored at −80 °C until assayed.

### 4.12. Protein Isolation and Kisspeptin Immunoblotting

To measure kisspeptin protein, the preoptic area (POA) and the mediobasal hypothalamus (MBH) were dissected with the aid of a precision brain slicer for adult mouse brain (ASI Instruments, Inc., Warren, MI, USA). The POA was defined as the region ventral to the anterior commissure extending rostrally from the caudal limit of the medial septum to the optic chiasm. The MBH was delineated rostrally by the posterior margin of the optic chiasm, laterally by the hypothalamic sulci, and caudally by the mammillary bodies. Tissue was homogenized in ice-cold radioimmunoprecipitation assay (RIPA) buffer [10 mM sodium pyrophosphate, 50 mM HEPES, pH 7.5, 1% NP-40, 50 mM NaCl, 50 mM NaF, 5 mM EDTA, 1 mM sodium orthovanadate, 1:1000 protease inhibitor cocktail (Sigma-Aldrich, USA), and 1 mM phenylmethanesulfonyl fluoride]. Protein lysates were stored at −80 °C until use. Total protein concentration was quantified via Bradford assay. Samples were run on 15% SDS-PAGE gels using a Mini-Protean Tetra Cell (BioRad, Hercules, CA, USA) and then transferred on to polyvinylidene difluoride (PVDF) membranes (0.2 um pore size, Immobilon-PSQ, Millipore-Sigma, USA). Membranes were blocked and incubated overnight with primary antibodies against kisspeptin (Abcam, Cambridge, MA, USA, ab19028, [63,64] and rabbit primary antibody against β-actin (Cell Signaling, 13E5) in 5% non-fat milk at 4 °C. Membranes were then probed with horseradish-peroxidase (HRP) anti-rabbit secondary antibodies (1:30,000, Cell Signaling, USA) for 1 h at room temperature. Membranes were incubated in enhanced chemiluminescence substrate West Dura Super Signal (Thermo Fisher Scientific, USA) and exposed on film. Film images were taken using a QICAM Fast 1394 digital camera (QImaging, Canada) and protein band densitometry for kisspeptin was performed using Image J software (National Institute of Health, Maryland, USA) normalized to β-actin as loading control.

### 4.13. Statistical Analyses

Data are expressed as mean ± SEM unless indicated otherwise. Statistical analyses were performed using Sigma Plot 10 (San Jose, CA, USA). For data on pubertal onset age, estrous cyclicity, testis weight, CL count, Kiss-GFP+ cell count, soma area, differences between WT and Kiss-PTEN KO mice were analyzed using unpaired Student’s t-test. For fertility rate data, statistical analysis was done using Fisher’s exact test. For experiments analyzing the effects of gonadectomy/steroid-hormone replacement, and 48-h fasting data were analyzed using a two-way ANOVA, followed by post-hoc Holm Šidák multiple comparisons method. GraphPad Prism 7 (Graph-Pad, San Diego, CA, USA) was used for graphic illustrations. For all statistical analyses, *p* < 0.05 was considered significant.

## Figures and Tables

**Table 1:** Reproductive physiology data grouped by genotype and sex.

Reproductive Phenotype	WT		Kiss-PTEN KO	
	Mean ± SEM	*n*	Mean ± SEM	*n*
**Females**				
Body weight (g)	19.4 ± 0.6	7	18.7 ± 0.8	8
VO age (d)	33 ± 1.3	9	34.0 ± 1.9	16
First estrus age (d)	44.5 ± 1.2	9	41.9 ± 1.1	16
Cycle length (d)	5.9 ± 0.6	5	5.3 ± 1.2	7
Mating Success (%)	83.3 ± 8.3	6	45.8 ± 11.9 *	6
Average Litter size	7.8 ± 0.4	6	5.5 ± 1.2	6
Number of CL/ovary	4.3 ± 0.9	4	4.2 ± 0.4	5
**Males**				
Body weight (g)	25.1 ± 0.6	6	23.2 ± 0.6	4
Testis weight (mg)	192 ± 8.9	7	188.0 ± 2.9	6
BS age (d)	36.2 ± 1.5	11	35.4 ± 0.9	19
Mating Success (%)	100 ± 0	3	100 ± 0	3

VO, vaginal opening, CL, corpora lutea, BS, balanopreputial separation, d, days. * Statistically significant difference (*p* < 0.05) when compared to WT group.

## Supplementary Materials

Supplementary materials can be found at https://www.mdpi.com/1422-0067/21/6/2107/s1.

## Abbreviations

ARC, arcuate nucleus of the hypothalamus; ArKO, aromatase KO; AVPV, anteroventral periventricular nucleus; CL, corporea lutea; DA, dopamine; E_2_, 17β estradiol; ERα, estrogen receptor alpha; 4E-BP1, eukaryotic initiation factor 4E-binding protein 1; FSH, follicle stimulating hormone; GDX, gonadectomy; GnRH, gonadotropin releasing hormone; HPG, hypothalamic-pituitary-gonadal; IHC, immunohistochemistry; ir, immunoreactivity; K_ATP_, ATP-sensitive potassium; KNDy, kisspeptin, neurokinin B and dynorphin expressing neurons; ME, median eminence, MBH, mediobasal hypothalamus; LH, luteinizing hormone; mTOR, mammalian target of rapamycin; NKB, neurokinin B; OVX, ovariectomy; POA, preoptic area; PTEN, Phosphatase and tensin homolog; PI3K, phosphatidylinositol 3 kinase; POMC, proopiomelanocortin; S6K1, S6 kinase 1; T, testosterone.
