# Supplementary material for: Targeted Deletion of PTEN in Kisspeptin Cells Results in Brain Region- and Sex-Specific Effects on Kisspeptin Expression and Gonadotropin Release
Source: Int J Mol Sci. 2020 Mar 19;21(6):2107. doi: 10.3390/ijms21062107 (PMC7139936; doi:10.3390/ijms21062107)
Supplement: Supplementary file 1 [file ijms-21-02107-s001.zip › ijms-720494 supplementary done/Supp Figure 1.pdf]

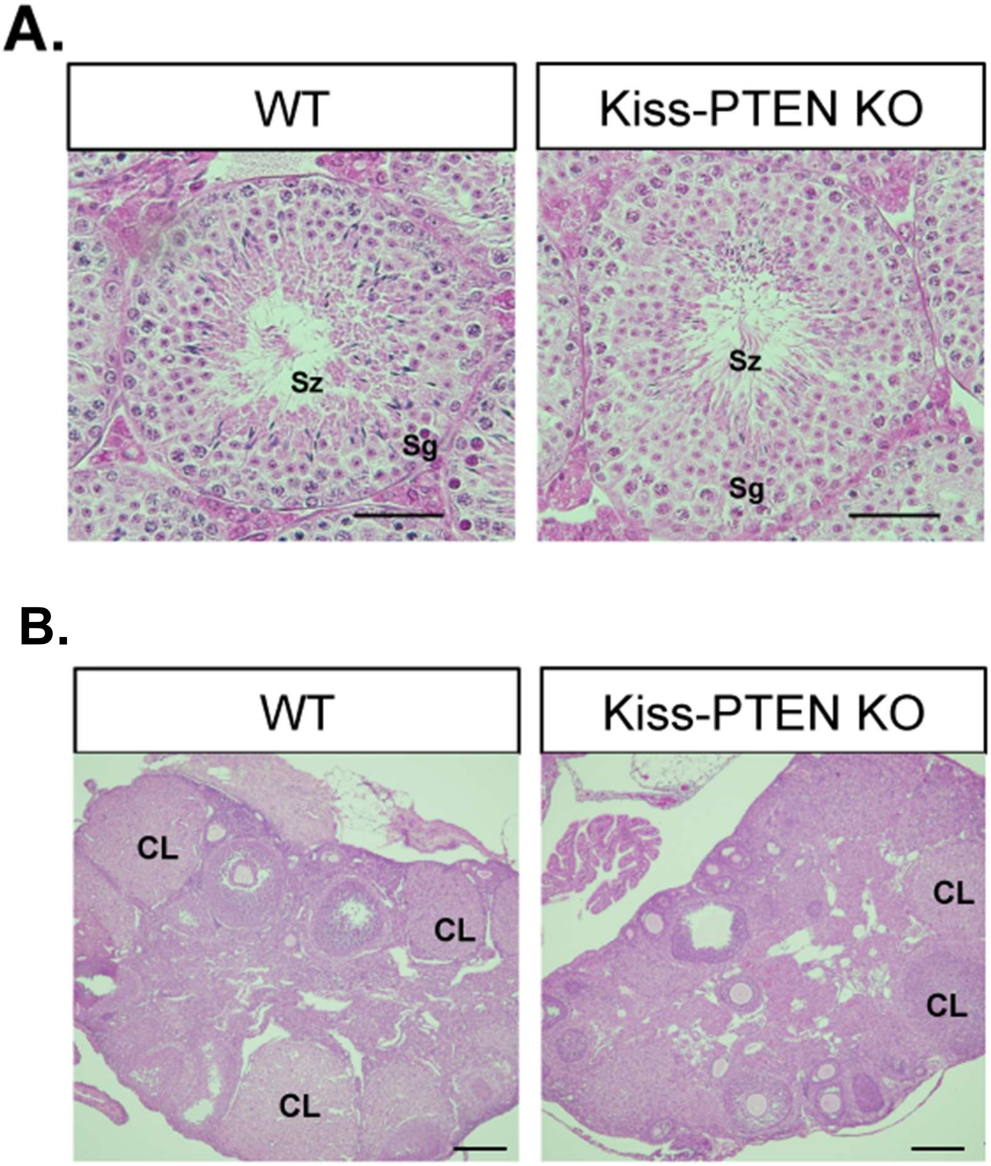

Supp. Fig. 1. (**A**) Representative photomicrographs of adult WT and Kiss-PTEN KO testicular tissue (cross-section) showing normal seminiferous tubules and spermatogonic cells (Sg, spermatogonia; Sz, spermatozoa). (**B**) Representative photomicrographs of adult WT and Kiss-PTEN KO ovaries (CL, corpus luteum). Scale bars: (**A**) = 50 µm; (**B**) = 200 µm.
