# Supplementary material for: Targeted Deletion of PTEN in Kisspeptin Cells Results in Brain Region- and Sex-Specific Effects on Kisspeptin Expression and Gonadotropin Release
Source: Int J Mol Sci. 2020 Mar 19;21(6):2107. doi: 10.3390/ijms21062107 (PMC7139936; doi:10.3390/ijms21062107)
Supplement: Supplementary file 1 [file ijms-21-02107-s001.zip › ijms-720494 supplementary done/Supp. Fig. 2 western.pdf]

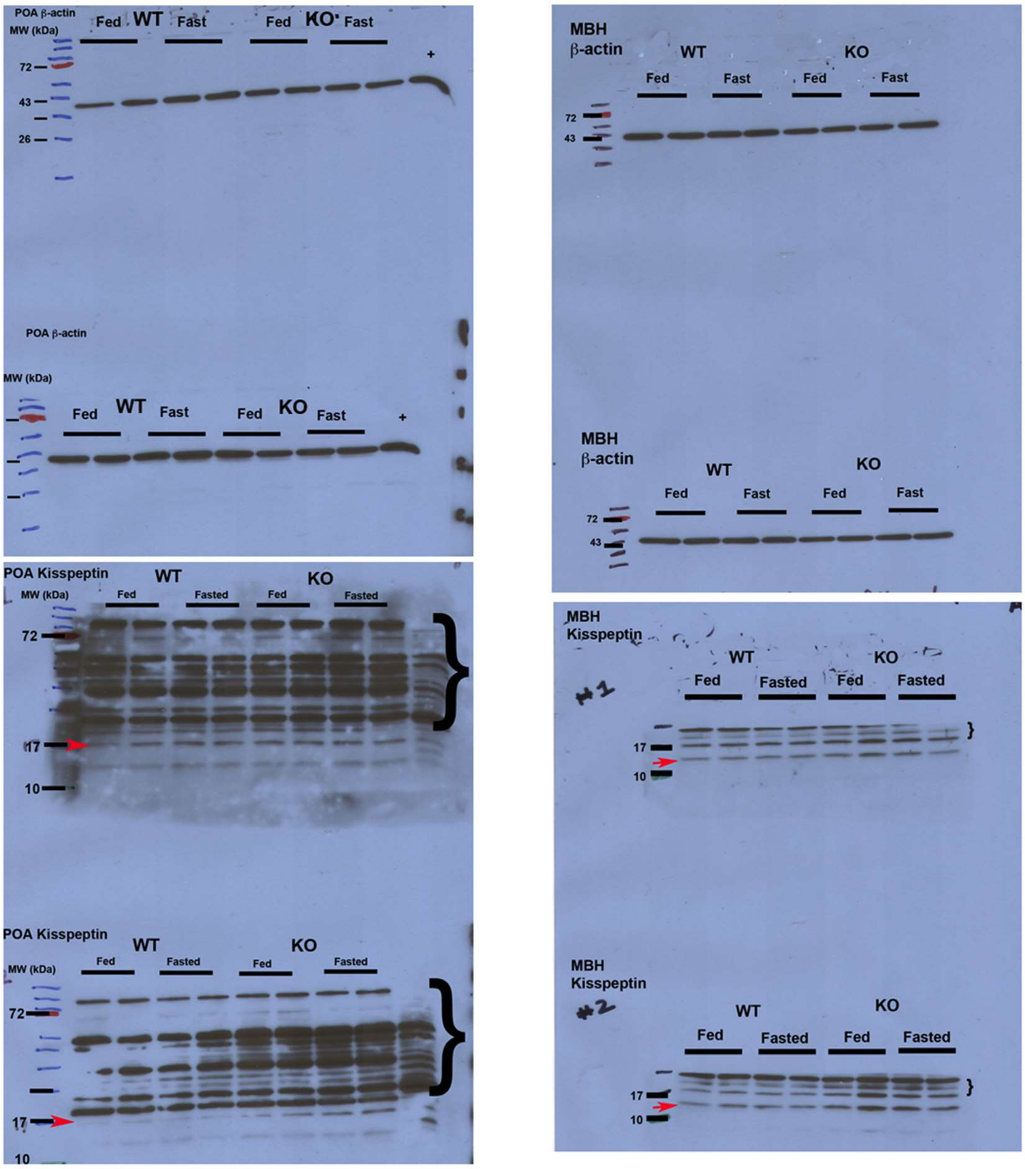

Supp. Fig. 2. Immunoblots showing detection of β-actin and kisspeptin protein (red arrow points to expected band at 15 kDa) in the POA and MBH of WT and Kiss-PTEN KO mice that were fed or fasted for 48 hours. Non-specific band are enclosed in brackets. MW, molecular weight markers.
