# Supplementary material for: Targeted Deletion of PTEN in Kisspeptin Cells Results in Brain Region- and Sex-Specific Effects on Kisspeptin Expression and Gonadotropin Release
Source: Int J Mol Sci. 2020 Mar 19;21(6):2107. doi: 10.3390/ijms21062107 (PMC7139936; doi:10.3390/ijms21062107)
Supplement: Supplementary file 1 [file ijms-21-02107-s001.zip › ijms-720494 supplementary done/Supp. Figure 3.pdf]

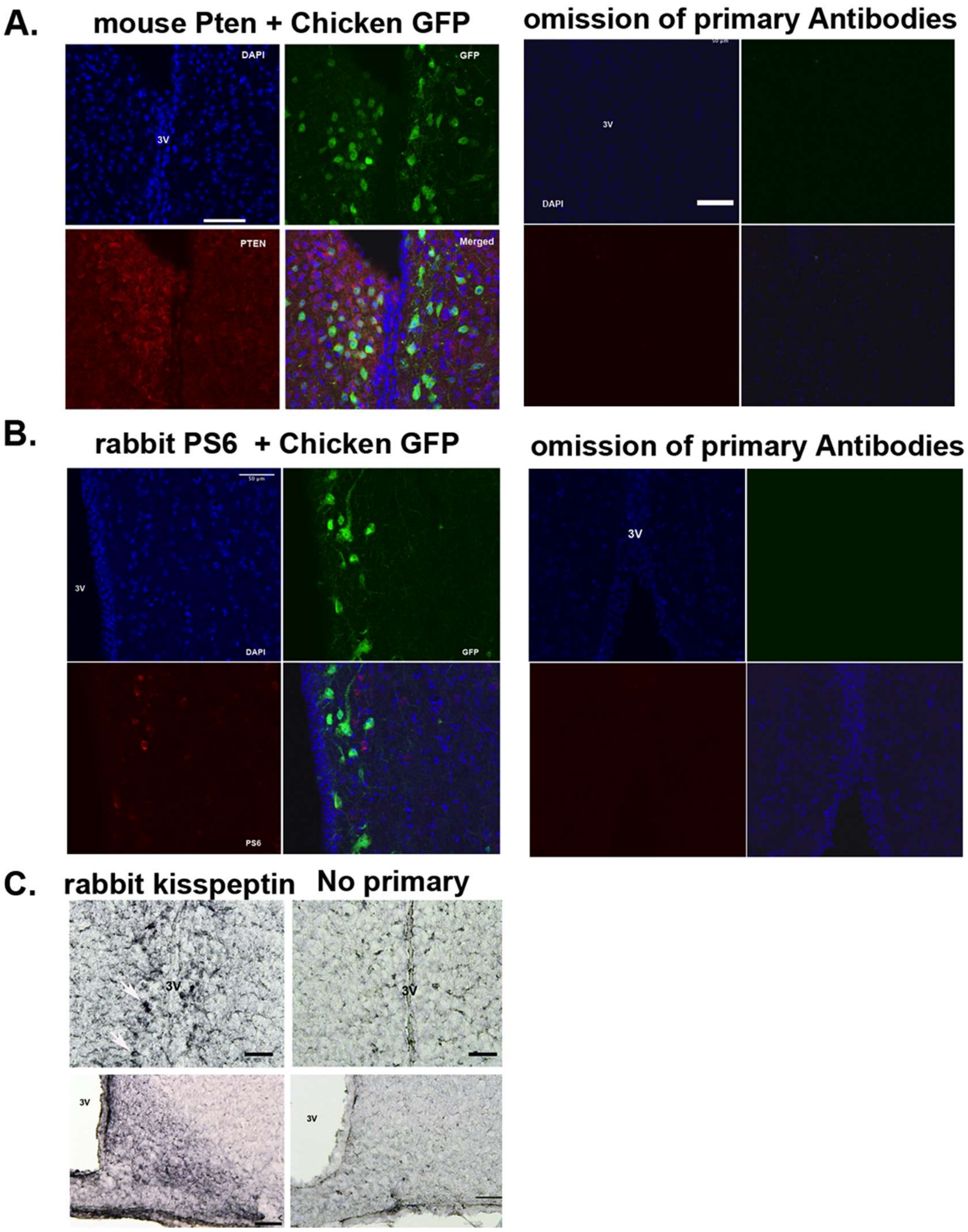

Supp. Fig. 3. (**A**) Representative immunofluorescent images of Kiss-GFP+ neurons (green), and Pten (red) in the AVPV with right panels showing representative images omitting the primary antibodies. (**B**) Representative immunofluorescent images of Kiss-GFP+ neurons (green), and pS6 (red), in the AVPV with right panels showing representative images omitting the primary antibodies. (**C**) Representative images of AVPV and ARC kisspeptin-ir with right panels showing representative images omitting the primary antibody.
