# Supplementary material for: Targeted Deletion of PTEN in Kisspeptin Cells Results in Brain Region- and Sex-Specific Effects on Kisspeptin Expression and Gonadotropin Release
Source: Int J Mol Sci. 2020 Mar 19;21(6):2107. doi: 10.3390/ijms21062107 (PMC7139936; doi:10.3390/ijms21062107)
Supplement: Supplementary file 1 [file ijms-21-02107-s001.zip › ijms-720494 supplementary done/Supp. Table 1.pdf]

Table 1. Primary antibody table

Peptide/protein target	Antigen sequence	Name of antibody	Manufacturer, catalog No.	Species raised in; monoclonal or polyclonal	Dilution used	References
phosphatase and tensin homologue deleted on chromosome ten (PTEN)	Carboxy-terminal sequence of human PTEN.	PTEN (26H9)	Cell Signaling, 9556S	Mouse; monoclonal	1:100	[42, 57, 58]
green fluorescent protein (GFP)	Purified recombinant GFP emulsified in Freund's adjuvant	Anti-GFP (green fluorescent protein)	AVES, GFP-1010	Chicken; polyclonal	1: 1000	
kisspeptin	N terminus of the full-length mouse KISS1 protein	Anti-kisspeptin	Abcam, ab19028	Rabbit; polyclonal	2 ug/mL	[63, 64]
kisspeptin	Ten amino acid C-terminal of murine kisspeptin (residues 43–52)	Anti-kisspeptin Batch #566	Alain Caraty, Institute National de la Recherche Agronomique, Paris, France	Rabbit; polyclonal	1:10,000	[55, 56]
β-actin	Amino-terminus of human β-actin	β-actin	Cell Signaling; 13E5	Rabbit; monoclonal	1:1000	
P70 S6 kinase	Residues surrounding (Ser235 and Ser236)	Phospho-S6 Ribosomal protein	Cell signaling, 2211S	Rabbit; polyclonal	1: 200	[59, 60]
